# Excess weight in children aged 2 to 5 years: crosssectional study to assess the discriminatory capacity of anthropometric indicators

**DOI:** 10.1590/1984-0462/2026/44/2025363

**Published:** 2026-11-13

**Authors:** Joelia Celeste Vieira Germano, Miriam de Andrade Brandão, Ana Carolina Patrício de Albuquerque Sousa, Mariana Porto Zambon

**Affiliations:** a Universidade Estadual de Campinas, Campinas, SP, Brazil.; b Universidade Federal do Rio Grande do Norte, Caicó, RN, Brazil.

**Keywords:** Public health, Anthropometry, Overweight, Pediatric obesity, Epidemiology, Saúde pública, Antropometria, Sobrepeso, Obesidade infantil, Epidemiologia

## Abstract

**Objective:**

To evaluate the association and discriminatory capacity of neck circumference (NC), waist circumference (WC), and waist-to-height ratio (WHtR) with excess weight, determining cutoff points.

**Methods:**

This cross-sectional study included 663 children (preschoolers aged 2 to 5 years) from the inland of northeastern Brazil. Sociodemographic (sex and age) and anthropometric (weight, height, NC, WC, and WHtR) variables were collected. Excess weight was defined by a body mass index-for-age Z score greater than +2 standard deviations. Increased NC and WC were defined as values above the 75th percentile of the sample. WHtR was considered elevated when ≥0.5. Descriptive and inferential statistics, analysis of diagnostic accuracy, and area under the receiver-operating characteristic (ROC) curve (AUC) were performed. The models were adjusted for anthropometric measurements, municipality, and/or age group. A significance level of 5% was adopted.

**Results:**

The findings showed an association (p<0.001) between NC and WC above the 75th percentile, and WHtR ≥0.50 with excess weight. The ROC curve analysis demonstrated that NC (AUC=0.903), WC (AUC=0.896), and WHtR (AUC=0.878) have good discriminatory capacity for identifying excess weight status in the age groups studied.

**Conclusions:**

In this study NC, WC, WHtR were associated with excess weight in preschoolers and also showed high discriminatory power.

## RESUMO

**Objetivo:**

Avaliar a associação e a capacidade discriminatória da circunferência do pescoço (CP), da circunferência da cintura (CC) e da relação cintura-altura (RCA) com o excesso de peso, determinando pontos de corte.

**Métodos:**

Trata-se de um estudo transversal com 663 crianças (pré-escolares de 2 a 5 anos de idade) do interior do Nordeste brasileiro. Foram coletadas variáveis sociodemográficas (sexo e idade) e antropométricas (peso, altura, CP, CC e RCA). O excesso de peso foi definido pelo escore Z do índice de massa corporal para idade superior a +2 desvios-padrão. A CP e a CC aumentadas foram estabelecidas com base em valores superiores ao 75º percentil da amostra. A RCA foi considerada elevada quando ≥0,5. Foram realizadas estatísticas descritivas e inferenciais, análise de acurácia diagnóstica e da área sob a curva (AUC) receiver operating characteristics (ROC). Foram realizados modelos ajustados por medidas antropométricas, município e/ou faixa etária. O nível de significância adotado foi de 5%.

**Resultados:**

Os achados evidenciaram associação (p<0,001) entre CP e CC acima do 75º percentil e RCA≥0,50 com excesso de peso. A curva ROC demonstrou que a CP (AUC=0,903), a CC (AUC=0,896) e a RCA (AUC=0,878) apresentam boa capacidade discriminatória para identificar excesso de peso nas faixas etárias estudadas.

**Conclusões:**

Neste estudo, CP, CC e RCA estiveram associadas ao excesso de peso em pré-escolares e apresentaram alto poder discriminatório.

## INTRODUCTION

 Excess weight is a global health issue,^1,2^ with the potential to undo advances that have contributed to increased life expectancy.^3,4^ Cases of obesity in children increase annually by 4.4%, higher than the 2.8% observed in adults.^5^

 Obesity modulates metabolic processes and inflammatory responses; therefore, adopting early prevention and intervention strategies to reduce the risk of chronic non-communicable diseases is essential.^2,6,7^

 During the growth and development process, children experience changes in the quantity and distribution of body components.^8 -10^ Assessing anthropometric measurements is important to monitor these changes, with the potential to contribute to the early identification of nutritional disorders.^10^ The scientific literature describes the identification of excess weight in preschoolers through standardized anthropometric indicators and multifactorial risk factors.^1-7^ The main diagnostic method is the use of growth charts that compare the child’s weight and height with reference populations.^3^ According to the World Health Organization (WHO) criteria, children are considered overweight if their Z scores are ≥ +2 standard deviations from the median reference body mass index (BMI) for their age, and not overweight if their Z scores are < +2 standard deviations from that median.^3^

 Although BMI is widely used for the initial screening of excess weight, it does not provide information on the distribution of body fat, which is considered more predictive of risk factors for chronic diseases.^11^ Therefore, it is recommended that the diagnosis of obesity in children and adolescents not be based solely on this index, since a more comprehensive assessment tends to be more accurate.^11-16^

 In this regard, indicators such as waist circumference (WC),^12^ neck circumference (NC),^13,14^ and waist-to-height ratio (WHtR),^15,16^ should be considered for greater specificity in assessing body adiposity.^9,10,12-17^ However, few studies examine the performance of these measures in identifying excess weight in preschoolers. 

 Considering the importance of assessing body fat distribution in obesity surveillance,^18^ the present study investigated the association and discriminatory capacity of NC, WC, and WHtR with excess weight in children aged 2–5 years, proposing cutoff points according to sex and age. 

## METHOD

 This study utilized data from the research “Evaluation of the prevalence and factors associated with childhood obesity”, approved by the Research Ethics Committee, under Approval No. 5,016,331 and CAAE No. 51663221.6.0000.5181. Participation was voluntary, and informed consent was provided by the guardians of the children through a Free and Informed Consent Form. 

 A cross-sectional study was carried out from October 2022 to November 2023 in two municipalities in northeastern Brazil: Caicó, in Rio Grande do Norte (Caicó-RN), and Patos, in Paraíba (Patos-PB). The main reason for choosing these cities was that both are located within the area of operation of the Multicampus School of Medical Sciences (EMCM) of the Federal University of Rio Grande do Norte, a co-participant in this research. The EMCM headquarters are located in Caicó-RN, but its influence extends throughout the hinterland of Rio Grande do Norte (RN) and Paraíba (PB). In addition to the undergraduate medical course, EMCM offers medical and multiprofessional residency programs, promoting teaching-service-community interaction in several municipalities in these two states. 

 Caicó is the eighth most populous city in Rio Grande do Norte, with 61,146 inhabitants, according to the last census. The Human Development Index (HDI) of the municipality is 0.71, while that of the state is 0.728. According to the United Nations Development Programme (UNDP), an HDI between 0.70 and 0.80 is considered high. Patos is the fourth most populous city in Paraíba, with an estimated population of 103,165 inhabitants. Patos’ HDI is 0.701, exceeding the state’s HDI of 0.658. Both municipalities have an educational system that includes daycare centers and schools linked to the Health in School Program, in addition to a health network almost 100% covered by Family Health Strategy teams. Daycare centers and Basic Health Units were the settings used for data collection in this study. 

 Sampling was conducted in a single stage using cluster sampling. Basic Health Units and daycare centers linked to the Health in School Program were selected as sampling units. The draw was conducted using the online platform https://www.random.org . 

 The study included preschoolers aged 2 years to 5 years. Children with endocrine or genetic diseases, –or other diseases that could compromise nutritional status or anthropometric measurements, as well as those with an incomplete assessment, were excluded from the study, as shown in Figure 1. 

**Figure 1:**
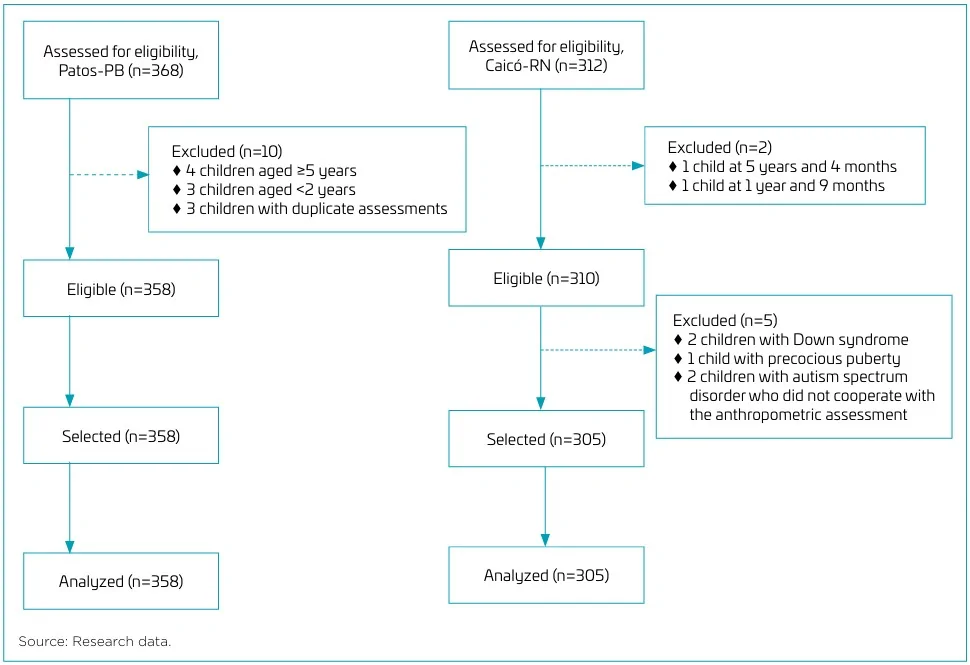
Flowchart of the study sample selection. Patos-PB and Caicó-RN, Brazil (2022–2023).

Source: Research data.

 For the sample size, the formula for infinite populations was used, considering the estimated prevalence of the event (p), the confidence level (Z), and the desired precision around the estimated prevalence (d^2^).^19^ Subsequently, the correction for finite populations was performed. Based on data from the Food and Nutrition Surveillance System, the estimated prevalence of overweight in children aged 2–5 years was 18.47% in Patos-PB, and 16.76% in Caicó-RN.^20^ The confidence level adopted was 95%, and the precision was 5%. 

 Based on the total population (4733 children in Patos-PB and 1902 in Caicó-RN), the initial estimated sample sizes were 221 for Patos-PB and 194 for Caicó-RN. Subsequently, after considering a design effect (deff) of 1.5 and a sample loss margin of 5%, the final estimated sample size was 655 children (349 in Patos-PB and 306 in Caicó-RN). 

 The data were obtained through interviews with legal guardians, using a questionnaire administered via Google Forms. The demographic characteristics analyzed included age and sex. For analysis, age was grouped into complete years. Anthropometry was performed following the techniques recommended by the Brazilian Ministry of Health.^21^

 Weight was measured using a calibrated portable electronic platform scale (model LS200P, Marte), with a capacity of 201 kg and an accuracy of 0.05 kg. Children stood barefoot in the center of the scale, wearing only underwear.^21^ To measure height, a wall-mounted stadiometer (model EST-221, Balmak) was used. It was fixed to a smooth wall without baseboards, with a measurement range of 0–220 cm and an accuracy of 0.1 cm. Children were positioned barefoot in the center of the stadiometer, with their heads free of props and aligned to the Frankfurt horizontal plane. Their heels, shoulders, and buttocks touched the wall, and their feet formed a right angle with the legs.^21^ The cutoff point definitions for BMI Z score classification were based on the criteria established by the WHO for children aged 0–5 years: severe thinness when BMI Z score < -3, thinness BMI Z score ≥ -3 and < -2, normal weight BMI Z score ≥ -2 and < +1, risk of overweight BMI Z score ≥ +1 and < +2, overweight when BMI Z score ≥ +2 and ≤ +3, and obesity when BMI Z score > +3.^22^ However, classifying as excess weight if Z BMI is ≥ +2 standard deviations from the reference median for age, and not excess weight if Z BMI is < +2 standard deviations from that median.^22^

 NC and WC measurements were obtained using a flexible, inelastic, millimeter-scale measuring tape with a 1.5 m extension. Children were positioned upright with their heads on the Frankfurt horizontal plane. 

 NC was measured at the mid-neck height, according to previously established protocol.^23^ No established cutoff points for diagnosing high NC in children aged 2–5 years were identified in the literature, and the 75th percentile of the sample was adopted as a reference to determine high values.^24^ The children were divided into two groups: those with NC ≤75th percentile (NC≤P75), and those with NC >75th percentile (NC>P75).^24^

 WC was measured at the midpoint between the last fixed rib and the upper edge of the iliac crest, at the end of a spontaneous expiration.^21^ To determine high WC values, the 75th percentile was used as a reference,^18^ considering parameters established by the Centers for Disease Control and Prevention.^25^ The children were divided into two groups: those with WC ≤75th percentile (WC≤P75) and those with WC >75th percentile (WC>P75).^18^

 The WHtR was calculated as waist circumference/height.^15,16^ The children were then categorized into two groups: those with WHtR <0.50 and those with WHtR ≥0.50.^16^

 To verify the normality of the data, the KolmogorovSmirnov test was used. To compare continuous variables between two groups, the Mann-Whitney test was used to analyze data expressed as median (25th–75th percentiles), and the independent sample t-test was used to analyze data presented as mean ± standard deviation. These variables included age, weight, height, BMI, Z BMI, NC, WC, and WHtR. 

 Pearson’s χ^2^ test or Fisher’s exact test were used to compare categorical variables between two groups. The variables analyzed included sex, age group (2, 3, and 4 years), prevalence of thinness, risk of overweight, overweight, and obesity, as well as the prevalence of high WC, high NC, and high WHtR. 

 Pearson’s χ^2^ test and Poisson regression were used to assess the association of NC, WC, and WHtR with excess weight. In Poisson regression, all analyses were conducted using the robust variance estimation method, with the results presented as prevalence ratio (PR) and 95% confidence intervals CI). Univariate and adjusted analyses were performed in Poisson regression to determine the association of anthropometric indicators with the outcomes. 

 For NC, the model was adjusted for WC, municipality, and age group. For WC, the model was adjusted for NC, municipality, and age group. For the WHtR, the model was adjusted only for municipality. The objective of these models was to verify whether the associations of NC and WHtR with excess weight, and of NC with elevated WC, remained statistically significant after adjusting for confounding factors. The analyses of the association of NC with excess weight and with elevated WC were stratified by sex. The analyses of WHtR with excess weight were performed for the total population, considering that this measure does not require adjustments for age and sex and is applicable to different ethnic groups.^16^

 The method used to assess the discriminatory capacity of NC, WC, and WHtR in identifying excess weight involved the analysis of diagnostic accuracy, including sensitivity, specificity, positive and negative predictive values (PPV, NPV), and area under the receiver operating characteristic (ROC) curve (AUC). The cutoff points for NC, WC, and WHtR were established based on the maximum Youden index points. Statistical analyses were conducted using Statistical Package for the Social Sciences (SPSS) for Windows, version 20.0 (IBM Corporation, Armonk, NY, USA), and MedCalc for Windows, version 22.021 (MedCalc Software, Ostend, Belgium). Statistical significance was set at a p<0.05. 

## RESULTS

 This study evaluated 680 children, 368 in Patos-PB and 312 in Caicó-RN. After excluding 10 children from Patos-PB and 7 from Caicó-RN, who did not meet the inclusion criteria, the final sample consisted of 663 children. 

 The proportions of boys and girls across municipalities did not differ significantly (p=0.90). Regarding age group, a higher proportion of 2-year-old boys was observed in Caicó-RN compared to Patos-PB (p=0.04). The medians of NC and WC were higher in boys from Patos-PB (p<0.001). Among girls, this difference was found only in the medians of NC and was also higher in Patos-PB (p=0.004). For the other variables, no significant differences were observed (Table 1). 

**Table 1.** Demographic and anthropometric characteristics of children aged 2–5 years. Patos-PB and Caicó-RN, Brazil.

Variables		Boys			Girls		
		Patos-PB (n=186)	Caicó-RN (n=160)	p-value	Patos-PB (n=172)	Caicó-RN (n=145)	p-value
Age, years*		3.59 (2.94–4.18)	3.38 (2.66–4.13)	0.211*	3.55 (2.82–4.01)	3.49 (2.87–4.12)	0.660*
Age range, years†							
	2	51 (27.40)	63 (39.40)		51 (29.70)	45 (31.00)	
	3	74 (39.80)	48 (30.00)	0.040†	77 (44.80)	55 (37.90)	0.411†
	4	61 (32.80)	49 (30.60)		44 (25.60)	45 (31.00)	
Weight, kg*		15.58 (14.00–17.80)	15.03 (13.55–17.40)	0.144*	15.05 (13.28–17.18)	15.15 (13.25–16.80)	0.798*
Height, cm*		99.50 (94.00–104.50)	97.20 (91.90–104.60)	0.123*	97.50 (91.50–102.00)	97.10 (91.70–103.20)	0.970*
BMI/age, kg/m^2^ *		16.10 (15.20–17.30)	15.90 (15.10–17.30)	0.620*	16.10 (14.80–17.50)	16.00 (15.10–17.30)	0.892*
Z BMI/age*		0.48 (-0.30–1.370)	0.37 (-0.38–1.24)	0.532*	0.49 (-0.34–1.42)	0.40 (-0.26–1.27)	0.881*
NC, cm*		25.00 (24.00–26.00)	24.10 (23.20–25.00)	<0.001*	24.50 (23.50–25.50)	23.50 (22.80–24.60)	<0.001*
WC, cm*		51.00 (48.50–53.00)	49.70 (47.50–51.90)	0.004*	50.00 (47.10–53.00)	49.50 (47.00–52.20)	0.144*
WHtR*		0.52 (0.49–0.54)	0.51 (0.49–0.54)	0.100*	0.52 (0.50–0.55)	0.51 (0.48–0.54)	0.152*

BMI/age: body mass index-for-age; Z BMI/age: body mass index-for-age Z score; NC: neck circumference; WC: waist circumference; WHtR: waist to-height ratio.

* Mann-Whitney test: data ar presented as median (25th–75th percentiles);

† Pearson’s χ^2^ test: data are presented as frequency (n) and proportions (%).

Source: Research data.

Table 2 presents the association between NC and WC categories and nutritional status (eutrophy, risk of overweight, and excess weight). Boys and girls with NC above the 75th percentile (>P75) had a higher risk of overweight and excess weight (p<0.001). Similarly, a WC above the 75th percentile (>P75) was associated with a higher prevalence of risk of overweight and excess weight in both sexes (p<0.001). 

**Table 2.** Association between neck and waist circumference with nutritional status (eutrophy, risk of overweight, and excess weight), in children aged 2–5 years. Patos-PB and Caicó-RN, Brazil.

		Boys (n=346)					Girls (n=317)				
		Eutrophy n (%)	Risk of overweight n (%)	Excess weight n (%)	χ^2^ (g)	p-value*	Eutrophy n (%)	Risk of overweight n (%)	Excess weight n (%)	χ^2^ (g)	p-value*
Neck circumference											
	≤P75	211 (79.90)	40 (15.20)	13 (4.90)	93.42(2)	<0.001	188 (75.80)	50 (20.20)	10 (4.00)	74.59(2)	<0.001
Waist circumference											
	≤P75	216 (84.40)	34 (13.30)	6 (2.30)	145.33(2)	<0.001	196 (83.80)	35 (15.00)	3 (1.30)	144.58(2)	<0.001

P: percentile; g: degrees of freedom;

* Pearson’s χ^2^ test: data presented as frequency (n) and proportions (%).

Source: Research data.

Table 3 describes the crude PR and adjusted (aPR) of excess weight, according to the anthropometric indicators analyzed. Even after adjustments, NC (p<0.001), WC (p<0.001), and WHtR (p<0.001) were all significantly associated with excess weight. 

**Table 3.** Prevalence of excess weight and crude prevalence ratios, according to anthropometric indicators (NC, WC, WHtR), in children aged 2–5 years. Patos-PB and Caicó-RN, Brazil.

	Boys (n=346)					Girls (n=317)				
	Not overweight n (%)	Excess weight* n (%)	p-value†	Prevalence ratio (95%CI)	p-value‡	Not overweigt n (%)	Excess weight* n (%)	p-value†	Prevalence ratio (95%CI)	p-value‡
NC≤P75	251 (95.10)	13 (4.90)	<0.001	1	<0.001	238 (96.00)	10 (4.00)	<0.001	1	<0.001
NC>P75	46 (56.10)	36 (43.90)		Crude PR 8.92 (4.97–15.98) § aPR 3.50 (1.94–6.33)		42 (60.90)	27 (39.10)		Crude PR 9.70 (4.94–19.05) § aPR 2.72 (1.47–5.01)	
WC≤P75	250 (97.70)	6 (2.30)	<0.001	1	<0.001	231 (98.70)	3 (1.30)	<0.001	1	<0.001
WC>P75	47 (52.20)	43 (47.80)		Crude PR 20.39 (8.98–46.27) / aPR 11.62 (4.85–27.87)		49 (59.00)	34 (41.00)		Crude PR 31.95 (10.08–101.27) / aPR 18.86 (5.95–59.76)	
Boys and Girls (n=663)											
WHtR<0.50	213 (99.10)	2 (0.90)	<0.001	1	<0.001					
WHtR≥0.50	364 (81.30)	84 (18.80)		Crude PR 20.16 (5.01–81.15) ¶ aPR 19.87 (4.93–80.08)						

PR: prevalence ratio; CI: confidence interval; NC: neck circumference; p: percentile; WC: waist circumference. WHtR: waist-to-height ratio;

* Excess weight: body mass index Z score ≥ +2;

† Pearson’s chi-square test: data presented as frequency (n) and proportions (%);

‡ Poisson regression with robust variance;

§ Adjusted for waist circumference, municipality, and age group;

/ Adjusted for neck circumference, municipality, and age group;

¶ Adjusted for municipality.

Source: Research data.

 To assess the diagnostic accuracy of anthropometric indicators in detecting excess weight, cutoff points were established and sensitivity, specificity, PPV, and NPV were assessed in children of different age groups and sexes (Table 4). 

**Table 4.** Cutoff points, sensitivities, specificities, positive predictive value and negative predictive value for anthropometric indicators (CC, WC, WHtR) associated with excess weight in children aged 2–5 years. Patos-PB and Caicó-RN, Brazil.

Age (years)			n	Cutoff (cm)	Youden index J	Sensitivity (95%CI)	Specificity (95%CI)	PPV (95%CI)	NPV (95%CI)
Neck circumference									
	Boys								
		2	114	>24.20	0.73	100.00 (79.40–100.00)	73.47 (63.60–81.90)	38.10 (30.70–46.10)	100.00
		3	122	>25.60	0.53	70.59 (44.00–89.70)	82.86 (74.30–89.50)	40.00 (28.40–52.90)	94.60 (89.20–97.30)
		4	110	>26.50	0.70	81.25 (54.40–96.00)	89.36 (81.30–94.80)	56.50 (40.90–71.00)	96.60 (91.00–98.70)
	Girls								
		2	96	>24.90	0.75	87.50 (47.30–99.70)	87.50 (78.70–93.60)	38.90 (25.70–54.00)	98.70 (92.50–99.80)
		3	132	>24.80	0.61	85.71 (57.20–98.20)	76.27 (67.60–83.60)	30.0 (22.50–38.70)	97.8 (92.60–99.40)
		4	89	>26.20	0.65	73.33 (44.90–92.20)	91.89 (83.20–97.00)	64.70 (44.50–80.70)	94.40 (88.00–97.50)
Waist circumference									
	Boys								
		2	114	>50.20	0.77	93.75 (69.80–99.80)	83.67 (74.80–90.40)	48.40 (37.00– 59.90)	98.80 (92.50–99.80)
		3	122	>53.00	0.77	88.24 (63.60–98.50)	89.52 (82.00–94.70)	57.70 (43.20–71.00)	97.90 (92.90–99.40)
		4	110	>57.50	0.84	87.50 (61.70–98.40)	96.81 (91.00–99.30)	82.40 (60.20–93.50)	97.80 (92.60–99.40)
	Girls								
		2	96	>51.70	0.92	100.00 (63.10–100.00)	92.05 (84.30–96.70)	53.30 (36.00–69.90)	100.00
		3	132	>55.50	0.85	85.71 (57.20–98.20)	99.15 (95.40–100.00)	92.30 (62.80–98.80)	98.30 (94.20–99.50)
		4	86	>56.50	0.83	86.67 (59.50–98.30)	95.95 (88.60–99.20)	81.20 (58.40–93.00)	97.30 (90.70–99.20)
Waist-to-height ratio									
	Boys and Girls								
			663	>0.54	0.60	75.58 (65.10–84.20)	84.40 (81.20–87.30)	41.90 (36.60–47.50)	95.90 (94.10–97.10)

CI: confidence interval; PPV: positive predictive value; NPV: negative predictive value.

Source: Research data.

 The diagnostic accuracy of the anthropometric indicators varied notably across age and sex, revealing distinct performance patterns for NC, WC, and WHtR (Table 4). For NC, the index demonstrated excellent screening capacity in the sample, achieving absolute sensitivity (100%) in 2-year-old boys and a balanced performance in 2-year-old girls (87.50%). However, NC diagnostic power tended to fluctuate and generally decrease with age, particularly regarding sensitivity in older children. 

 In contrast, WC emerged as a highly robust and stable predictor for both sexes across all age groups; it consistently maintained high sensitivity and specificity, peaking in specificity among 3-year-old girls (99.15%) and 4-year-old boys (96.81%). This suggests that WC is a more dependable cross-sectional marker for early childhood assessment than NC (Table 4). 

 Finally, the generalized WHtR cutoff point (>0.54) for children aged 2–5 years provided a moderate-to-high overall diagnostic profile, yielding a higher capability to correctly rule out excess weight (specificity of 84.40%) than to detect it (sensitivity of 75.58%) (Table 4). 

## DISCUSSION

 The primary finding of this study is the robust discriminatory capacity of NC, WC, and WHtR as screening tools for excess weight in preschoolers. Notably, the results demonstrate that NC maintains a significant association with overweight and obesity even after rigorous adjustment for age, municipality, and, crucially, WC. This suggests that NC provides independent diagnostic value, capturing aspects of adiposity that traditional abdominal measures might overlook. 

 While WC exhibited superior accuracy specifically among 3- and 4-year-old girls, the overall performance of these indicators with AUC values consistently exceeding 0.89 positions them as highly reliable proxies for adiposity in early childhood. 

 The observation that WHtR≥0.5 was associated with a nearly 20-fold increase in the prevalence ratio for excess weight (aPR=19.87) further underscores the critical nature of these thresholds in identifying high-risk pediatric profiles. These findings are in line with studies carried out in Cypriot children aged 6–10 years^17^ and Pakistani children aged 5–14 years,^13^ although the study designs in these investigations differed slightly from the methodology used in the present study. 

 Moreover, a critical appraisal of the data reveals that NC performance (AUC=0.903) outperformed findings by Hu et al. (AUC=0.822).^26^ This discrepancy is likely not accidental; rather, it reflects the methodological refinement in utilizing narrower, one-year age intervals (e.g., 2–3, 3–4, 4–5 years) compared to the broader brackets used in prior literature. This granularity allows for a more precise alignment with the rapid physiological growth phases inherent in the 2–5-year age group. 

 When compared to studies in Turkey^14^ and Pakistan,^26^ the findings of the present study highlight how outcome definition significantly alters diagnostic sensitivity. The sensitivity and specificity of NC varied from 70.59–85.71% and from 76.2791.89%, respectively, according to age group and sex. These values are close to those obtained in similar studies^14,26^, and the differences observed can be attributed to variations in the population characteristics and methodologies adopted in each study. By evaluating "excess weight" (encompassing both overweight and obesity) rather than obesity alone, this study provides a more inclusive screening threshold. This is a strategic choice: in a public health context, identifying the early stages of weight gain is more valuable for preventive intervention than identifying established obesity. 

 For WC, the results indicated a significant association with overweight, even after adjustments for confounding factors. In addition, the ROC curve, with values consistently >0.896, shows good discriminatory capacity. These findings corroborate other studies conducted with Brazilian children (4–7 years),^27^ Thai children (5–6 years),^28^ and Dutch children (3–7 years).^29^ Notably, in the present study, the AUCs were calculated separately by sex and age group, which may provide a more accurate assessment. 

 The analysis of the WC cutoff points showed high sensitivity and specificity in detecting excess weight. For children aged 2–5 years of both sexes, the sensitivity, specificity, PPV, and NPV values were consistent, corroborating other studies,^28^ suggesting that the ability of WC to identify overweight children may be robust in different population contexts and age groups. Regarding WHtR, values ≥0.5 were significantly associated with excess weight, even after adjustments for confounding factors (aPR=19.87), and this association was confirmed by AUC=0.878, corroborating those reported by Vieira et al.^27^ and Leone et al.^30^

 The ROC curves of NC and WC were compared to detect excess weight, and no statistically significant differences were observed in most analyses. However, for 3- and 4-year-old girls, WC demonstrated greater accuracy, suggesting that it may be a stronger indicator of excess weight than NC in certain age groups and sexes. No similar studies were found in the age groups investigated in this study, indicating a gap in the literature. 

 The implications of these findings for the pediatric population of northeastern Brazil and similar developing regions are profound. Traditional methods of assessing body composition, such as Dual-energy X-ray Absorptiometry (DXA) or even standardized BMI-for-age, can be logistically challenging or culturally sensitive in primary care settings. However, despite the strength of the associations, the cross-sectional nature of this study limits the ability to establish temporal causality between the increase in NC/WC and long-term metabolic outcomes. Additionally, while the evaluators were rigorously trained, the inherent risk of measurement bias in anthropometry cannot be eliminated. Future longitudinal research is essential to track how these indicators evolve alongside the child’s development and to validate these cutoff points across diverse ethnic and socioeconomic cohorts. 

 Finally, this study highlights that the anthropometric measures NC, WC, WHtR were associated with excess weight in preschoolers and showed high discriminatory power. NC and WC are non-invasive, low-cost, and culturally acceptable. NC avoids the need to undress the child, which may increase compliance during routine screenings. By establishing valid cutoff points for children as young as 2 years old, this study empowers health professionals to detect abnormal weight trajectories before they transition into chronic metabolic conditions in adolescence. 

## Data Availability

The database that originated the article is available with the corresponding author.
